# Gene network inference from single-cell omics data and domain knowledge for constructing COVID-19-specific *ICAM1*-associated pathways

**DOI:** 10.3389/fgene.2023.1250545

**Published:** 2023-08-31

**Authors:** Mitsuhiro Odaka, Morgan Magnin, Katsumi Inoue

**Affiliations:** ^1^ The Graduate University for Advanced Studies, SOKENDAI, Tokyo, Japan; ^2^ Principles of Informatics Research Division, National Institute of Informatics, Tokyo, Japan; ^3^ Laboratoire des Sciences du Numérique de Nantes, École Centrale de Nantes, Nantes Université, UMR 6004, Nantes, France; ^4^ Japan Society for the Promotion of Science, Tokyo, Japan

**Keywords:** ICAM-1, cell-to-cell transmission, COVID-19, single-cell omics data analysis, model validation, pathway enrichment analysis, data-driven and knowledge-based approach

## Abstract

**Introduction:** Intercellular adhesion molecule 1 (ICAM-1) is a critical molecule responsible for interactions between cells. Previous studies have suggested that ICAM-1 triggers cell-to-cell transmission of HIV-1 or HTLV-1, that SARS-CoV-2 shares several features with these viruses via interactions between cells, and that SARS-CoV-2 cell-to-cell transmission is associated with COVID-19 severity. From these previous arguments, it is assumed that ICAM-1 can be related to SARS-CoV-2 cell-to-cell transmission in COVID-19 patients. Indeed, the time-dependent change of the ICAM-1 expression level has been detected in COVID-19 patients. However, signaling pathways that consist of ICAM-1 and other molecules interacting with ICAM-1 are not identified in COVID-19. For example, the current COVID-19 Disease Map has no entry for those pathways. Therefore, discovering unknown *ICAM1*-associated pathways will be indispensable for clarifying the mechanism of COVID-19.

**Materials and methods:** This study builds *ICAM1*-associated pathways by gene network inference from single-cell omics data and multiple knowledge bases. First, single-cell omics data analysis extracts coexpressed genes with significant differences in expression levels with spurious correlations removed. Second, knowledge bases validate the models. Finally, mapping the models onto existing pathways identifies new *ICAM1*-associated pathways.

**Results:** Comparison of the obtained pathways between different cell types and time points reproduces the known pathways and indicates the following two unknown pathways: (1) upstream pathway that includes proteins in the non-canonical NF-*κ*B pathway and (2) downstream pathway that contains integrins and cytoskeleton or motor proteins for cell transformation.

**Discussion:** In this way, data-driven and knowledge-based approaches are integrated into gene network inference for *ICAM1*-associated pathway construction. The results can contribute to repairing and completing the COVID-19 Disease Map, thereby improving our understanding of the mechanism of COVID-19.

## 1 Introduction

Elucidating the underlying mechanisms of coronavirus disease 2019 (COVID-19) still remains a global issue. Uncovering the mechanism requires fully understanding the COVID-19-specific interactome, a complex network of interactions among different components. In previous studies on COVID-19, attempts to provide insights into interactions within cells have been made. In contrast, interactions between cells have not been closely examined.

One noteworthy molecule responsible for the interactions between cells is intercellular adhesion molecule 1 (ICAM-1; also known as CD54), encoded by *ICAM1*. ICAM-1 is a transmembrane glycoprotein expressed on leukocytes, vascular endothelial cells, and respiratory epithelial cells. Its differential expression is critical for proinflammatory immune responses and viral infection. Additionally, ICAM-1 enables interactions between cells by controlling leukocyte migration, homing, and adhesion from outside to inside the cell (outside-in) and regulation from inside to outside the cell (inside-out) (Luo et al., 2007). These functionalities make ICAM-1 an attractive drug target and a clinically essential molecule (Wilson et al., 1993). Another premise regarding ICAM-1 as an essential molecule in this study is grounded by a hypothesis on the interaction between cells, called cell-to-cell transmission. Cell-to-cell transmission is a direct viral transfer from one cell to another cell (Igakura et al., 2003). The previous model-driven study suggested that compared to the other viral transfer manner called cell-free transmission, cell-to-cell transmission would be more associated with COVID-19 severity (Odaka and Inoue, 2021). In fact, cell-to-cell transmission is observed in *in vitro* experiments involving COVID-19’s pathogen, severe acute respiratory syndrome coronavirus 2 (SARS-CoV-2) (Zeng et al., 2021). Moreover, cell-to-cell transmission is observed in other retroviruses, such as human immunodeficiency virus type 1 (HIV-1) and human T-cell leukemia virus type 1 (HTLV-1), whose functionalization is similar to that of SARS-CoV-2 (Walls et al., 2020). Specifically, both these retroviruses and SARS-CoV-2 have a structurally homologous spike glycoprotein on the surface of the viral envelope that binds to a surface protein on the recipient cell during cell adhesion (Weissenhorn et al., 1999). In HIV-1 or HTLV-1, the cell-to-cell transmission occurs after ICAM-1 triggers the peculiar pathways for cell adhesion (Bracq et al., 2018) and induces the formation of the microtubule-organizing center (MTOC) and virological synapse (VS) (Nejmeddine et al., 2009). The aforementioned arguments provide a rationale for focusing on ICAM-1 in this study and for hypothesizing the *in vivo* existence of ICAM-1 and the interactions between cells featured with ICAM-1 involved in cell-to-cell transmission in COVID-19.

Indeed, there have been different *in vitro* experimental results on the expression level of ICAM-1 in SARS-CoV-2-infected cells. One study shows the time-dependent ICAM-1 expression level changes in COVID-19 patients (Smith-Norowitz et al., 2021). Another study also shows that the ICAM-1 level increases in the severe phase and decreases in the convalescent phase of COVID-19 (Tong et al., 2020). Another study shows the opposite result on the decrease of ICAM-1 after immune cell infiltration in COVID-19 while leaving room for controversy regarding the reasons for downregulation (Won et al., 2022).

Nevertheless, the interactions arising from ICAM-1 are not explicitly recognized as indispensable in the case of COVID-19. In particular, there is little insight into the signaling pathways surrounding ICAM-1, that is, the upstream and downstream signaling cascades that occur upon the functional activation of ICAM-1 and its specific signaling molecules interacting with ICAM-1 (i.e., *ICAM1*-associated pathways for short). Consequently, it is significant to uncover the *ICAM1*-associated pathways to better understand the interactions between cells in the context of COVID-19.

Another substantial consequence of revealing *ICAM1*-associated pathways contributes to completing the COVID-19 Disease Map. The COVID-19 Disease Map is a graphical knowledge repository based on pathway enrichment analysis or manual curation from external knowledge bases, such as the Kyoto Encyclopedia of Genes and Genomes (KEGG) pathways (Ostaszewski et al., 2019). The COVID-19 Disease Map increases the pathway volume; however, it is indicated that the map lacks genes without pathway annotations (Reimand et al., 2019). Moreover, the initial version of the COVID-19 Disease Map was built on the fly (Ostaszewski et al., 2020), so it has inherently been incomplete and in progress. As for ICAM-1, the pathways and even ICAM-1 are absent in the current COVID-19 Disease Map (Ostaszewski et al., 2021). Thus, this study regards it challenging to find unknown *ICAM1*-associated pathways, expecting these pathways to include the molecules driving cell-to-cell transmission.

Given the aforementioned observations, this study constructs the *ICAM1*-associated pathways based on gene networks. For inferring gene networks, we harness data and domain knowledge by extracting relationships between gene pairs from data while rectifying them with multiple knowledge bases. Such integration of data-driven and knowledge-based approaches allows us to avoid biologically meaningless interpretations based only on data characteristics. Identifying the unknown pathways with biologically meaningful interpretation will lead to a deeper understanding of the mechanisms of COVID-19.

## 2 Materials and methods

### 2.1 Overview

**FIGURE 1:**
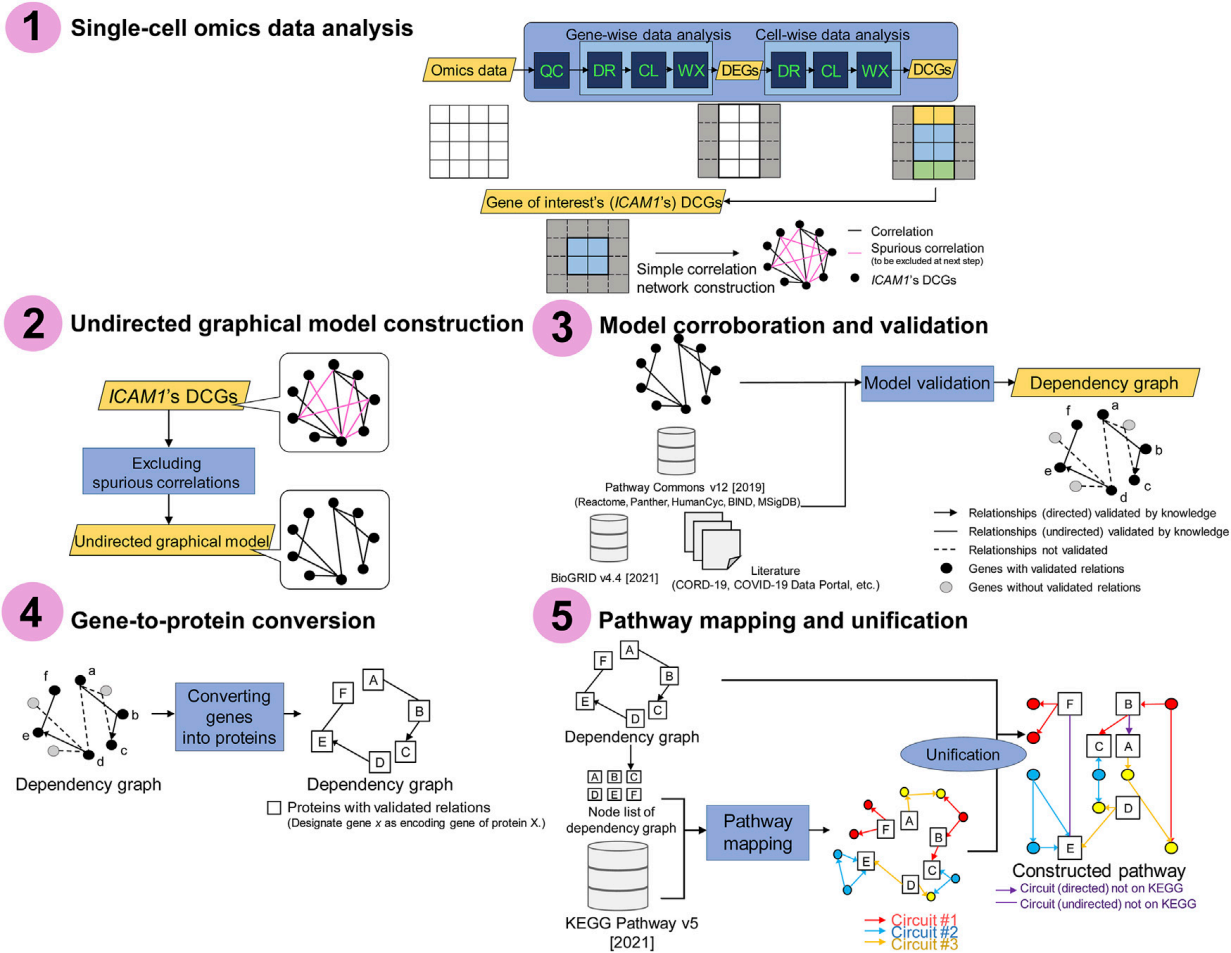
Schematic representation of the framework. Step 1: single-cell omics data analysis. Step 2: undirected graphical model construction. Step 3: model corroboration and validation. Step 4: gene-to-protein conversion. Step 5: pathway mapping and unification. The *circuits* are subpathways transmitting a signal from input receptor nodes to output effector nodes, where the nodes mostly represent proteins such as metabolic enzymes. QC: quality control; DR: dimensionality reduction; CL: clustering; WX: Wilcoxon rank-sum test; DEGs: differentially expressed genes; DCGs: differentially coexpressed genes. See also DOI: 10.6084/m9.figshare.18095717.

Figure 1 illustrates the framework of this study. This framework constructs the disease-specific pathways from single-cell omics data and domain knowledge via gene network inference. The framework consists of the following five steps:

1. Single-cell omics data analysis
2. Undirected graphical model construction
3. Model corroboration and validation
4. Gene-to-protein conversion
5. Pathway mapping and unification

Steps 1 and 2 are dedicated to gene network inference purely from data, and Step 3 validates the data-driven gene network with domain knowledge. In this study, we call the rectification of data-driven objects with knowledge a data-driven and knowledge-based (DD-KB) approach. Step 1 involves obtaining the COVID-19-specific differentially expressed genes (DEGs) and a network of differentially coexpressed genes (DCGs) via single-cell omics data analysis. Here, DEGs are the genes whose expression levels differ significantly in COVID-19-positive patients and -negative controls, and DCGs are coexpressed DEGs. Step 2 removes spurious edges from the correlation networks, thereby building *de novo* undirected graphical models. In corroboration (Step 3), undirected graphical models are edited as dependency graphs with validated relationships.

Steps 4 and 5 are pathway construction steps. In Step 4, a functional annotation tool is used to convert genes into encoded proteins. Pathway mapping and unification (Step 5) refine the results as the final outputs, the *ICAM1*-associated pathways. Through the framework, single-cell omics data and multiple knowledge bases are integrated, which allows the inference of gene networks containing the components absent from the current COVID-19 Disease Map.

### 2.2 Gene network inference and pathway construction

In this subsection, explanations for each step of the framework are provided.

#### 2.2.1 Step 1: Single-cell omics data analysis

Single-cell omics data analysis adopts a combination of the standard methods defined as three subroutines, i.e., dimensionality reduction, clustering, and Wilcoxon rank-sum test, for each gene pair and each cell pair (Li et al., 2017). This step can extract COVID-19-specific DEGs and *ICAM1*-associated DCGs from the omics data.

##### 2.2.1.1 Dimensionality reduction

Dimensionality reduction is executed after the imputation of zeros representing either technically missing data or biologically absent genes within a matrix of single-cell omics data (Hicks et al., 2018). To reduce dimensionality, we employ two methods: principal component analysis (PCA) and uniform manifold approximation and projection (UMAP). These methods detect possible batch effects and embed the matrix in the latent space. By computing 50 PCA coordinates on the sparse matrix for mean centering (Pedregosa et al., 2011), eigenvalues, and eigenvectors with the singular value decomposition solver ARPACK (ARnoldi PACKage) (Lehoucq et al., 1998), PCA reduces the dimension to 100 by a Gaussian kernel. Given the 50 decomposed coordinates, the connectivities (weighted adjacency matrix) of the *k*-nearest neighborhood graph are computed and thresholded at the closest neighbors defined for data points of the manifold in Euclidean space. Following PCA, UMAP (McInnes and Healy, 2018) projects the data points onto the two-dimensional latent space.

##### 2.2.1.2 Clustering

Afterward, clustering is enforced to classify data points in the latent space into subgroups by similarity measurements and filtering out the genes unassociated with the gene of interest. The Louvain algorithm, a greedy optimization of local modularity to detect the groups (Blondel et al., 2008), is applied for clustering. Clustering allows obtaining the data points of subgroups with similar gene expression profiles.

##### 2.2.1.3 Wilcoxon rank-sum test

The Wilcoxon rank-sum test is conducted to sort the data points and select the top 200 data points within a cluster. This test compares the signal values between each subgroup and the combination of the other subgroups using the Benjamini–Hochberg method for adjusting the false discovery rate and correcting the *p*-value (Benjamini and Hochberg, 1995). The comparison allows us to detect significant differences in expression levels between COVID-19-positive and COVID-19-negative patients and rank the genes characteristic of each subgroup.

The aforementioned analysis, including dimensionality reduction, clustering, and Wilcoxon rank-sum test, is conducted for each gene pair and each cell pair. Genewise analysis filters the DEGs to distinguish those whose gene expression levels are correlated. Here, given that functionally related genes are coexpressed in the same clusters, the identified gene clusters can be considered to include the genes with significant differences in expression levels from the negative control, and the genes within the same cluster share a common differential expression pattern (Eisen et al., 1999). Likewise, cellwise analysis filters the DCGs to classify all the cells into cell clusters based on the correlation coefficients as similarity measurements for embedding, which means that the genes within the same cell cluster are more strongly correlated with each other than with the genes in other clusters. Constraining the DCGs with the gene of interest, *ICAM1*, provides a subset of DCGs correlated with *ICAM1*.

#### 2.2.2 Step 2: Undirected graphical model construction

Next, we infer gene networks from *ICAM1*’s DCGs obtained by the single-cell omics data analysis. The gene networks are inferred as undirected graphical models with a partial correlation method, displaying de novo-produced direct linear associations (de la Fuente et al., 2004). Considering that correlated gene pairs are coexpressed with similar functions, designating any gene pair as nodes and the correlation coefficient of gene expression levels as edges forms the simple correlation networks of *ICAM1*’s DCGs. Calculating the second-order partial correlation coefficients between all gene pairs and removing the edges of the gene pairs with zero partial correlation coefficients for any combination yield undirected graphical models without spurious correlations (Zuo et al., 2014).

The equations for the zero-order, first-order, and second-order partial correlations are shown in Eqs 1, 2, 3, respectively.

$$Zero–order\;correlation:r_{xy}=\frac{cov\left(xy\right)}{\sqrt{var\left(x\right)var\left(y\right)}},$$ (1)

$$First–order\;partial\;correlation:r_{xy,z}=\frac{r_{xy}-r_{xz}r_{yz}}{\sqrt{\left(1-r_{xz}^{2}\right)\left(1-r_{yz}^{2}\right)}},$$ (2)

$$Second–order\;partial\;correlation:r_{xy,zq}=\frac{r_{xy,z}-r_{xq,z}r_{yq,z}}{\sqrt{\left(1-r_{xq,z}^{2}\right)\left(1-r_{yq,z}^{2}\right)}}.$$ (3)

The random variables denoted by *x*, *y*, *z*, and *q* represent the gene names. *r*_*xy*_ is Pearson’s correlation coefficient between the gene expression-level vector running over all the cells of any gene *x* and that of any gene *y*. The simple correlation network starts by connecting *x* and *y* if and only if *r*_*xy*_ ≠ 0. Undirected graphical modeling removes the linear effect of all the second-order partial correlation coefficients *r*_*xy*,*zq*_ between two variables (*x*, *y*) conditional on all other variables. The edge is weighted as ${\left(0.5+0.5\cdot r_{x,y}\right)}^{12}$ to follow the scale-free law, which typically holds for a weighted gene coexpression network (WGCN) (Langfelder and Horvath, 2008).

#### 2.2.3 Step 3: Model corroboration and validation

Until the previous data analysis, gene networks consisting of the *ICAM1* gene of interest are inferred without guaranteeing the validity of each edge. Namely, possible errors within data, such as noise, could result in nodes or edges with no biological meaning. Hence, the models require corroboration and validation with heuristics based on domain knowledge. To corroborate and validate each relationship of gene networks, we query multiple knowledge bases, including Pathway Commons Web Service 12 (Rodchenkov et al., 2020), BioGRID REST Web Service (Stark et al., 2006), and STRING version 11.5 (Szklarczyk et al., 2019). Pathway Commons’ application programming interface (API) provides access to the significant pathway databases Reactome, PANTHER, HumanCyc, BIND, and MSigDB. BioGRID is used as a complementary source of the latest knowledge since Pathway Commons is not up-to-date (Wadi et al., 2016). HumanCyc is used because it has richer information on biochemical reactions and regulatory relationships than the KEGG pathways alone (Altman et al., 2013) and enables the obtained model to include more information than a subset of the KEGG pathways. STRING is used for annotations of functional or physical interactions between the queried proteins. Fetching relations between gene pairs in the simple interaction format (SIF) through these knowledge bases enables us to convert a subset of undirected edges to directed edges, thereby editing undirected graphical models as dependency graphs.

The subsequent two steps are dedicated to the pathway construction by overlaying the inferred DD-KB gene networks onto the KEGG pathways.

#### 2.2.4 Step 4: Gene-to-protein conversion

There exists a gap between the gene network and the KEGG pathways because the nodes of the DD-KB gene networks are DCGs (genes), while the nodes of the KEGG pathways are primarily proteins. Therefore, this gap needs to be filled before overlaying the DD-KB gene networks onto the KEGG pathways. The DAVID functional annotation tool 6.8 (Huang et al., 2009b; Huang et al., 2009a) allows us to fill the gap by converting gene symbols into Entrez IDs. We apply the DAVID tools to the node lists of the DD-KB gene networks to give the corresponding protein attributes for each DCG.

#### 2.2.5 Step 5: Pathway mapping and unification

In order to examine what types of pathways are activated, we conduct pathway enrichment analysis by mapping the protein node lists and edge lists of the DD-KB gene networks onto the KEGG pathways. In particular, the DD-KB gene networks and the KEGG pathways in the KEGG Markup Language (KGML) format are unified using Cytoscape 3.9.0 (Shannon et al., 2003), resulting in the final COVID-19-specific *ICAM1*-associated pathways, visualized in yFiles Hierarchical Layout.

### 2.3 Application

We applied the aforementioned framework to the two COVID-19 datasets for comparing the *ICAM1*-associated pathways between different locations where *ICAM1* is expressed (case study 1) and between different time points starting from hospitalization (case study 2).

#### 2.3.1 Case study 1: Comparison of *ICAM1*-associated pathways between different cell types

**FIGURE 2:**
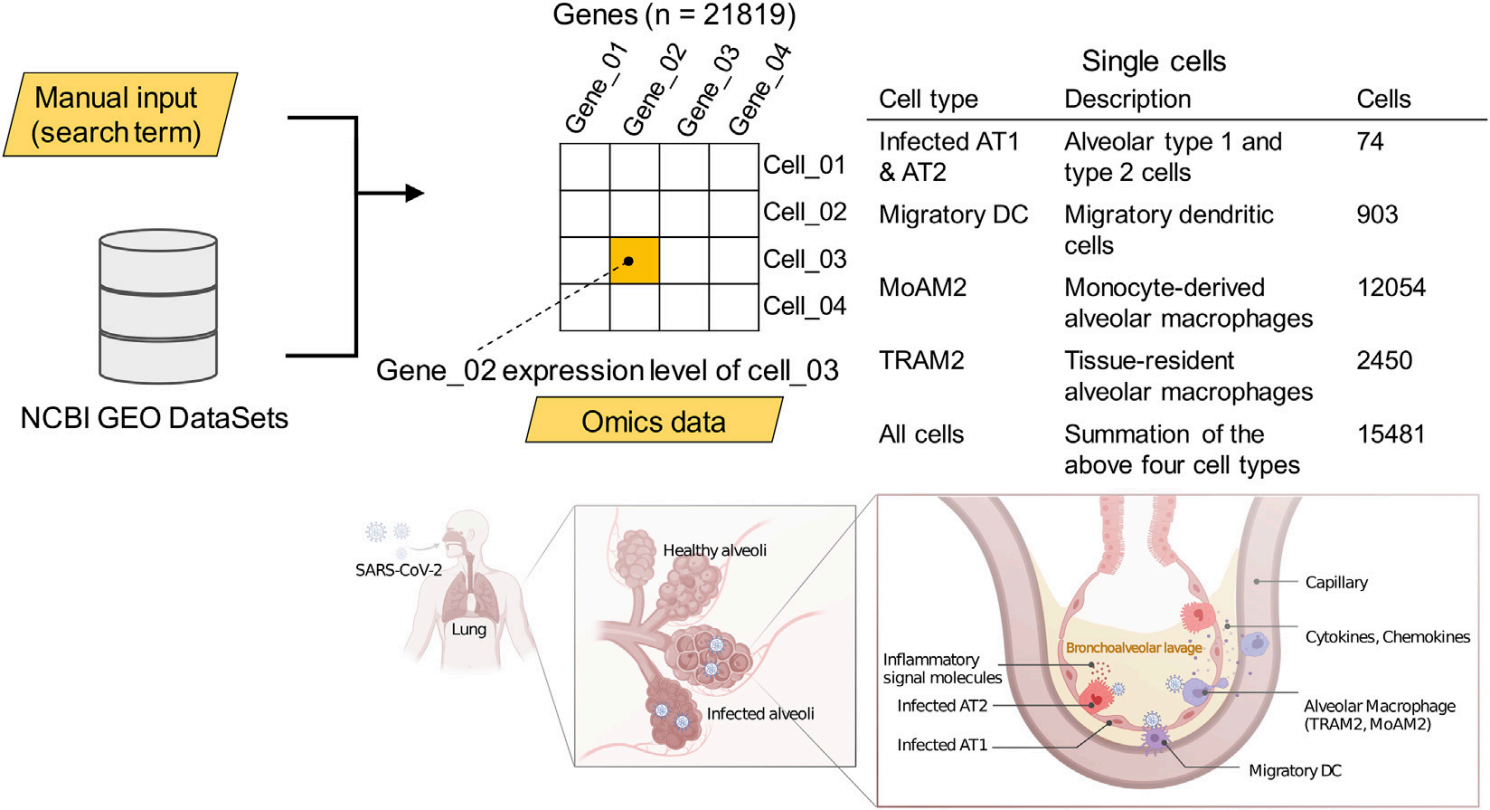
The cell types for which data were collected. Pulmonary tissue illustrations: created with BioRender.com. See also DOI: 10.6084/m9.figshare.18095714.

Inputting the search term [(COVID-19 OR SARS-CoV-2) AND gse(entry type)] AND “*Homo sapiens*” AND h5ad to the National Center for Biotechnology Information (NCBI) Gene Expression Omnibus (GEO) dataset (Barrett et al., 2013) provided the data. In case study 1, the data included the gene expression profiles of bronchoalveolar lavage fluid samples isolated from 10 patients with severe COVID-19 and two negative controls via high-throughput single-cell RNA sequencing (Grant et al., 2021). Especially, we used the data of messenger ribonucleic acid (mRNA) expression levels from four antigen-presenting cell types in which virus particles were detectable. The single-cell omics data analysis in the original paper had already annotated the cell types, cell subpopulation partitioning the cell heterogeneity into non-overlapping classes, for clusters according to the reference biomarkers present in the cluster (Duò et al., 2018). The cell types included infected alveolar type 1 and 2 cells (infected AT1 and AT2), migratory dendritic cells (migratory DCs), tissue-resident alveolar macrophage type 2 (TRAM2), and monocyte-derived alveolar macrophage type 2 (MoAM2), as well as the summation of these four cell types at the level of full single-cell resolution (Figure 2).

#### 2.3.2 Case study 2: Comparison of *ICAM1*-associated pathways between different time points

Likewise, GSE180578, another transcriptome omics dataset, was (Cillo et al., 2021) fetched from the NCBI GEO dataset. The omics data contained 86 samples obtained by single-cell RNA sequencing, including peripheral blood from COVID-19 patients or negative control at the intensive care unit (ICU) of the University of Pittsburgh Medical Center. These samples included three time points (days 1, 5, and 10 post-enrollment in the ICU). The cell counts and gene counts were (34970, 2000), (23616, 2000), and (32105, 2000).

#### 2.3.3 Additional case studies

Not limited to *ICAM1*, our framework would be reusable in another context for mixing quantitative data and domain knowledge into building models capturing pathways. To allege the generality of the framework, in case study 2, we also applied the framework to other genes related to the interaction between cells, including ACTB and C15orf48. Here, in this main manuscript, we focus only on providing the results of *ICAM1*-associated pathways considering the purpose of this study. The results of other genes can be referred to in the Supplementary Material.

### 2.4 Quality control

Before proceeding to the further steps, the single-cell omics data underwent quality control. Quality control included filtering, scaling, and normalization by Scanpy version 1.8.2 (Wolf et al., 2018). Given the cell quality, we regarded the cells with overexpressed mitochondrial RNA per data count tagged by a unique molecular identifier (UMI) (Kivioja et al., 2012) as dead or broken cells. Similarly, cells with many genes per data count tagged by UMI were identified as doublets. Subsequently, the genes detected in fewer than three cells were filtered out to ensure gene quality. The count data were scaled with regression on total UMI counts and normalization per feature based on standard deviation. Normalization of the gene expression data was adjusted for the RNA composition bias and allowed a comparison of the values among the cells. Finally, log-transformation prepared the data for calculating the log-fold changes reflecting the gene expression difference.

The framework was applied concerning the two datasets. The machine configuration was as follows: Python 3.7, GPU Tesla V100-SXM2-16GB, and 51.01 GB of RAM.

## 3 Results

### 3.1 Case study 1: *ICAM1*-associated pathways at different locations (cell types)

#### 3.1.1 Quality control

The data initially contained 77,650 cells × 24,714 genes. Removing the cells with a high proportion of mitochondrial RNA resulted in 15,220 cells. After filtering, the whole dataset contained 68,734 cells × 24,001 genes. Among this dataset, we used the data of four cell types, 21,819 genes in the SARS-CoV-2-infected 15,481 cells (cells with SARS-CoV-2 transcripts detected). The doublet discrimination provided 14,723 cells. The filtering processes excluded 8,916 cells with more than 5,000 expressed genes, 700 genes detected in fewer than three cells, mitochondrially encoded genes, and cells with a low percentage (<10%) of mitochondrial genes, leaving 17,644 genes. Cells with less than one gene count were filtered out, leaving 9,050 cells. The quality control ultimately yielded log-transformed normalized gene expression data for 9,050 cells × 17,644 genes.

#### 3.1.2 Single-cell omics data analysis

The genewise analysis extracted the 18 gene clusters with differential expression patterns specific to COVID-19. Excluding the duplicated genes extracted 1,434 DEGs in 9,050 single cells. The results of genewise clustering, heatmap of DEGs, and rank-sum test are shown in Supplementary Figures S1–S3, respectively. The cellwise analysis yielded 11 clusters based on the correlations between gene pairs in the embedded space and distinguished the DEGs whose gene expression levels were correlated. One of the 11 clusters included *ICAM1*, and this cluster was made of 178 *ICAM1*’s DCGs. The results of cellwise clustering, heatmap of DCGs, and rank-sum test are shown in Supplementary Figures S4–S6, respectively. The entire list of DEGs and DCGs can be found in Supplementary Tables S1, S2, respectively. Supplementary Table S1 is a hash table of DEGs, including the cluster number, gene name string, log fold change, and *p*-values, while Supplementary Table S2 is a hash table of DCGs, including gene expression levels for each single cell. The *p*-values of *ICAM1* expression variation and the computation times (sec.) for each cell type are as follows: *p* = 0.250, time = 51.6 (infected AT1 and AT2); *p* = 2.50E-4, time = 26.1 (migratory DC); *p* = 2.57E-12, time = 46.6 (TRAM2); *p* = 7.55E-2, time = 109.0 (MoAM2); and *p* = 0.241, time = 178.9 (summation).

#### 3.1.3 Undirected graphical model construction

Removal of spurious correlations yielded undirected graphical models (Table 1). The finally obtained undirected graphical models are shown in Supplementary Figure S7.

**TABLE 1:** Spurious correlation removal in case study 1.

Cell types	Nodes	Edges (full)	Edges (excluded)	Edges (output)
Infected AT1 and AT2	116	6670 (100%)	6296 (94%)	374 (6%)
Migratory DCs	248	30628 (100%)	28695 (94%)	1933 (6%)
TRAM2	150	11175 (100%)	8690 (78%)	2485 (22%)
MoAM2	152	11476 (100%)	7819 (68%)	3657 (32%)
Summation	179	15931 (100%)	12529 (79%)	3402 (21%)

The table depicts the number of nodes, the number of edges in the simple correlation network (full model), the number of spurious edges removed by calculating the second-order partial correlation coefficients, and the number of ultimately left edges.

#### 3.1.4 Model corroboration and validation

Dependency graphs are shown in Supplementary Figure S8. The entire list of relationships of dependency graphs with knowledge bases used for model validation can be found in Supplementary Table S3.

#### 3.1.5 Gene-to-protein conversion

The nodes in the dependency graphs were annotated with protein names, which helped us map the nodes onto the KEGG pathways in the next step.

#### 3.1.6 Pathway mapping and unification

Pathway mapping discovered which subpathways within existing signaling pathways reflect the activity of a group of varying genes and coexpressed in a disease-specific manner in the observed gene expression data. Table 2 shows the typical pathways selected from the mapping results. A complete list of mapping results is shown in Supplementary Figure S9.

**TABLE 2:** Mapping results of the dependency graphs for each cell type onto the KEGG pathways.

Cell types	Scores	Matched KEGG pathways
Infected AT1 and AT2	0 genes (no match)	
Migratory DCs	40 genes (63.5% match)	NF-*κ*B signaling pathway (hsa04064) (12) and HTLV-1 infection (hsa05166) (9)
TRAM2	23 genes (65.7% match)	Influenza A (hsa05164) (13) and HTLV-1 infection (hsa05166) (5)
MoAM2	31 genes (54.4% match)	NF-*κ*B signaling pathway (hsa04064) (3)
Summation	18 genes (64.3% match)	TNF signaling pathway (hsa04668) (3) and NF-*κ*B signaling pathway (hsa04064) (3)

Scores count the “matched” genes on the dependency graphs, whose encoding proteins are found on any of the KEGG pathways and their proportion to total gene counts. Matched KEGG pathways exemplify how many matched genes are included in a specific pathway. For example, if gene *x*’s encoded protein *X* is on KEGG pathways A and B, one is added to the score, and both A and B are represented.

**FIGURE 3:**
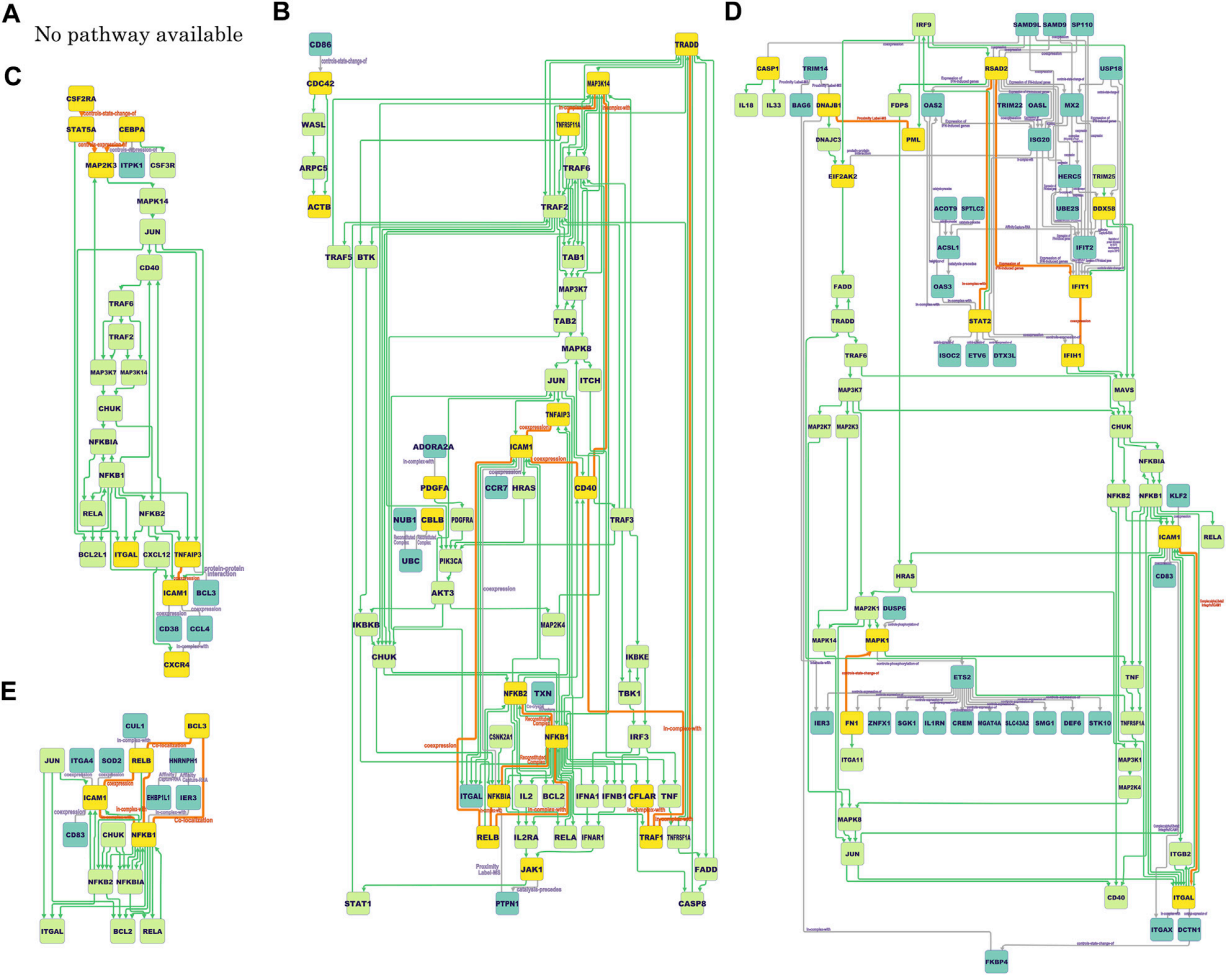
*ICAM1*-associated pathways at different locations (cell types). **(A)**: No pathway available (infected alveolar type 1 and 2 cells); **(B)**: NF-*κ*B/non-canonical NF-*κ*B/integrin pathway putative (migratory dendritic cells); **(C)**: NF-*κ*B/integrin pathway putative (tissue-resident alveolar macrophages); **(D)**: NF-*κ*B/integrin pathway putative (monocyte-derived alveolar macrophages); **(E)**: TNF/NF-*κ*B/non-canonical NF-*κ*B/integrin pathway putative (summation). The rectangular nodes colored blue, yellow, and lime green reflect the proteins only on the dependency graphs, the proteins common to both the dependency graphs and the KEGG pathways, and the proteins only on the KEGG pathways, respectively. Gray lines are the directed or undirected edges only on the dependency graphs. Orange lines represent the directed or undirected edges between yellow nodes on the dependency graphs. Green lines are the directed edges only on the KEGG pathways. Orange edges do not have direction if the KEGG pathways indirectly connect its yellow node pair. See also DOI: 10.6084/m9.figshare.17261540.

The final COVID-19-specific *ICAM1*-associated pathways for each cell type are shown in Figure 3. Although pathway mapping was performed by converting gene symbols to protein IDs before mapping, the nodes of pathways in the figure are assigned only gene symbol names for space limitation. For the *ICAM1*-associated pathway for infected AT1 and AT2 (Figure 3A), there are no hits among the KEGG pathways, which is attributed to only one pair of validated *ICAM1*-associated DCGs that remained.

The characteristics common to the obtained pathways and the characteristics of those pathways for each cell type are as follows: one common feature of the pathways for all other cell types except Figure 3A is the presence of some integrins, such as ITGAL (gene encoding CD11a; also known as LFA1A) (Figures 3B–E), ITGAX (gene encoding CD11c) (Figure 3D), ITGB2 (gene encoding CD18) (Figure 3D), and ITGA4 (gene encoding CD49d) (Figure 3E). Some integrins are downstream, such as ACTB (gene encoding *β*-actin) in the pathway for migratory DCs (Figure 3B) and DCTN1 (gene encoding dynactin subunit 1) in the pathway for MoAM2 (Figure 3D). Integrins are molecules interacting with ICAM-1 to stabilize cell adhesion. Especially, dynactin recruits and tethers dynein to microtubules.

Another common feature of the pathways for all other cell types except Figure 3A is the presence of the molecules responsible for NF-*κ*B pathways, such as NFKB1 (gene encoding NF-*κ*B p105 subunit 1), NFKB2 (gene encoding NF-*κ*B p105 subunit 2), RELA (gene encoding NF-*κ*B p65 subunit), JUN (Jun proto-oncogene; also known as AP-1 transcription factor subunit), and CHUK (gene encoding inhibitor of nuclear factor *κ*-B kinase subunit *α*; also known as IKK-*α*) (Figures 3B–E). These molecules are not DCGs but nodes in the KEGG pathway, but they are located upstream of *ICAM1* and flanked by DCGs.

As the specific features of some pathways, the pathway for migratory DCs (Figure 3B) and summation (Figure 3E) includes RELB (gene encoding transcription factor RelB). In general, NFKB2 and RELB lie in the non-canonical NF-*κ*B pathway, which is an upstream pathway of *ICAM1* (Liu et al., 2019). The pathway for migratory DCs (Figure 3B) also includes TNFRSF11A (gene encoding receptor activator of NF-*κ*B; also known as RANK) and MAP3K14 (gene encoding NF-*κ*B-inducing kinase; also known as NIK). These molecules are known as triggers of the non-canonical NF-*κ*B pathway (Malinin et al., 1997).

The pathway for summation (Figure 3E) has some commonalities with other pathways, such as integrins and molecules related to the NF-*κ*B pathway, but it also has some differences. SOD2 (gene encoding superoxide dismutase 2), for example, is a gene that is not found in the other pathways and could not be found without taking summation. SOD2 is known as a gene whose expression variation has been confirmed accompanied with *ICAM1* in COVID-19 (Zheng et al., 2021).

### 3.2 Case study 2: *ICAM1*-associated pathways at different time points

Like case study 1, the original dataset underwent single-cell omics data analysis, and *ICAM1* and its DCGs were extracted. The *p*-values of *ICAM1* expression variation and the computation times (sec.) for each time point were as follows: *p* = 1.50E-05, time = 77.9 (day 1); *p* = 3.09E-2, time = 55.4 (day 5); and *p* = 3.04E-06, time = 56.3 (day 10). This manuscript does not explain the other detailed results of the single-cell omics data analysis because the procedures were the same as that in case study 1. These other results can be found at 10.6084/m9.figshare.23590755. As for the remaining steps, we explain the results of spurious correlation removal and pathway construction.

#### 3.2.1 Undirected graphical model construction

Table 3 depicts how spurious correlated edges were removed for each of the three time points. Of the number of edges in the simple correlation network (full model), more than 84% of the edges were deleted by calculating the second-order partial correlation coefficients.

**TABLE 3:** Spurious correlation removal in case study 2.

Day	Nodes	Edges (full)	Edges (excluded)	Edges (output)
1	121	7,503 (100%)	6,309 (84%)	1,194 (16%)
5	198	20,706 (100%)	18,914 (91%)	1,792 (9%)
10	126	8,001 (100%)	6,748 (84%)	1,253 (16%)

#### 3.2.2 Pathway mapping and unification

**FIGURE 4:**
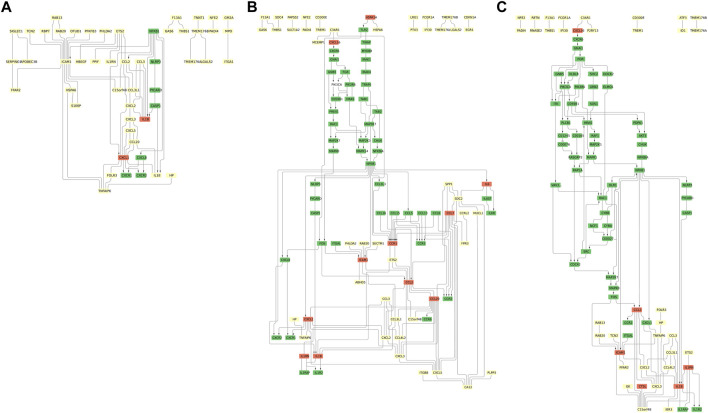
*ICAM1*-associated pathways at different time points. **(A)** NF-*κ*B/MAPK pathway putative (day 1). **(B)** NF-*κ*B/MAPK pathway putative (day 5). **(C)** NF-*κ*B/MAPK pathway putative (day 10). The light yellow, green, and orange nodes represent data-driven DCGs, the genes listed only in the KEGG pathways, and the genes derived from both data and the KEGG pathways, respectively. The directed edges are the edges whose directions are given in the KEGG pathways. See this figure for a larger view in figshare: 10.6084/m9.figshare.23576226.

The pathways resulting from combining the KEGG pathways with the partial correlation networks of *ICAM1*-associated DCGs extracted from the omics data are shown in Figure 4.

The common characteristics and the unique attributes of the found pathways for each time point are as follows: one common feature is the presence of molecules responsible for the immune response included across the three time points. The immunoreactive molecules include the chemokine ligand (*CXCL1, 2, 3*) induced by interleukin-1 (*IL1B*) or TNF-*α*-induced protein 6 (*TNFAIP6*). These molecules are along the green-colored molecules, such as the NF-*κ*B p105 subunit (*NFKB1*) and NLRP3 inflammasome (*NLRP3*, *PYCARD*, and *CASP1*), which are related to proinflammatory effects and activation of the NF-*κ*B pathway or the MAPK pathway (Bouwmeester et al., 2004). Other common molecules include the modulator of cytochrome C oxidase during inflammation (*C15orf48*), chemokine ligands acting as macrophage inflammatory protein (*CCL3* and *CCL3L1*), transmembrane protein (*TMEM176A/B*), nuclear factor erythroid (*NFE2*), peptidyl arginine deiminase 4 (*PADI4*), RAS oncogene (*RAB20*), and proto-oncogene (*ETS2*). These are also related to inflammation, immune response, or membrane fusion (Pylayeva-Gupta et al., 2011; Gold et al., 2012; Tanikawa et al., 2012; Lee et al., 2021).

As the specific features of two pathways, the pathways at days 1 and 5 include pleckstrin homology-like domain family A member 2 (*PHLDA2*) and chemokine ligand (*CXCL5*) (Figures 4A, B). The pathways at days 5 and 10 include chemokine ligands acting as macrophage inflammatory protein (*CCL4L2*) (Figures 4B, C). The pathways at days 1 and 10 include folate receptor (*FOLR3*), haptoglobin (*HP*), and chemokine ligand (*CXCL16*) (Figures 4A–C). These are related to acute inflammatory response, immunity, or membrane attachment (Palomino and Marti, 2015; Cohen et al., 2020; Duan et al., 2022).

The obtained pathways do not contain the molecules in the NF-*κ*B pathway (*NFKB1*, *NFKBIA*, and *CHUK*) or the MAPK pathway (*HRAS*, *RAF1*, and *MAP2K7*). The absence of these molecules may be due to insufficient gene coverage in the original omics data.

## 4 Discussion

### 4.1 *ICAM1*-associated pathways from case studies

The comparison between the obtained *ICAM1*-associated pathways in case study 1 with the COVID-19 Disease Map reveals existing and unknown nodes. For example, MAP2K3, MAPK14, JUN, FOS, ITGA2, ITGB1, RSAD2, OAS, and STAT2 have already been mapped onto the COVID-19 Disease Map, while RELB, ITGAL, CDC42, ACTB, CD40, DCTN1, BCL3, and CD83 in the obtained pathways are still absent in the current COVID-19 Disease Map.

Likewise, we can identify the difference between the obtained *ICAM1*-associated pathways and the current COVID-19 Disease Map from the results of case study 2. For instance, *IL1B*, *NFKB1*, *NLRP3*, *PYCARD*, and *CASP1* are listed in the COVID-19 Disease Map, while there are molecules absent from it, including *CCL3, 3L1, 4L2*, *CXCL1, 2, 3, 5, 16*, *TNFAIP6*, *C15orf48*, *TMEM176A/B*, *NFE2*, *PADI4*, *RAB20*, *ETS2*, *PHLDA2*, *FOLR3*, and *HP*.

The results from both case studies indicated that the NF-*κ*B pathway would likely be activated, which reflects that our framework can reproduce the already-known fact that the NF-*κ*B pathway is activated in COVID-19, as seen in the KEGG’s COVID-19 pathway (hsa05171). As a new insight into the unknown pathways is missing from the current COVID-19 Disease Map, the results imply that COVID-19 involves the following two up/downstream pathways:

• Upstream pathway with proteins on the non-canonical NF-*κ*B pathway
• Downstream pathway with integrins and cytoskeletal elements associated with actin and the motor protein dynein for cell transformation

The non-canonical NF-*κ*B pathway is reasonable because it is relevant to the proinflammatory response in viral infections such as COVID-19. It is also creditable that TNFRSF11A is found only in the pathway of DCs (Figure 3B) since TNFRSF11A is known to be expressed on DCs and T cells to facilitate their interaction with each other (Anderson et al., 1997). The involvement of downstream pathways leading to the cytoskeleton (the internal filaments of eukaryotic cells), including actin filaments and microtubules, in COVID-19 is also plausible. After the interaction between ICAM-1 and integrin regulates cell adhesion, the motor protein myosin would move on actin filaments, inducing cell transformation and movement. The motor protein dynein would move on microtubules transporting molecules in the cytoplasm to the MTOC. Given the argument mentioned in the Introduction that MTOC or VS spawned by ICAM-1 causes cell-to-cell transmission in HIV-1 or HTLV-1, the existence of these downstream molecules of *ICAM1*-associated pathways raises the possibility of pathways involved in the formation of MTOC or VS in SARS-CoV-2. In this study, the Ras-Raf-MEK-ERK pathway for MTOC or VS in HTLV-1 was inactive. Meanwhile, RAC1 and CDC42 were conserved. Ras-related C3 botulinus toxin substrate 1 (Rac1, encoded by RAC1) and cell division control protein 42 homolog (Cdc42, encoded by CDC42) are essential for VS formation in HTLV-1 cells (Gross and Thoma-Kress, 2016). Although it is unclear whether SARS-CoV-2 has a VS formation mechanism analogous to that of HIV-1 or HTLV-1, we cannot rule out the possibility that MTOC formation and VS formation never occur. To verify these inferred phenomena, observing MTOC and VS formation through infection experiments or molecular dynamics tracking using high-end live-cell imaging techniques (Real et al., 2018) would be desirable.

### 4.2 Related work

The need to identify unknown pathways has accelerated the work related to gene network inference in COVID-19. For example, Hasankhani et al. (2021) obtained signaling pathways associated with the main hallmarks of COVID-19 by differential coexpression network analysis. Tanaka et al. (2021) revealed host cellular gene networks by using the Bayesian network. Generally, several methods for gene network inference from single-cell omics data exist, which can be classified into data-driven and knowledge-based methods. Data-driven gene network inference methods include statistical approaches such as regression, mutual information, correlation, and a combination of different techniques (Marbach et al., 2012; Mochida et al., 2018). Alternatively, knowledge-based gene network inference uses prior knowledge for information retrieval or logic programming. Fabris et al. quantified the influence by creating interpretable KEGG feature types for the hierarchical classification of aging-related protein functions (Fabris and Freitas, 2016). Chen et al. provided the biological relevance by analyzing the Gene Ontology terms and KEGG pathways of each drug category enriched in the literature and clinical trials for predicting the drug–target interaction (Chen et al., 2015). There also exist hybrid methods incorporating data-driven and knowledge-based methods. Soh et al. enumerated the minimal network components by adopting a Boolean satisfiability problem (SAT) solver for KEGG pathways (Soh et al., 2012). Zuo et al. integrated information at gene expression and network topology levels by differentially weighted graphical LASSO (Zuo et al., 2017). However, full-scale integration of data-driven and knowledge-based methods is still under development for gene network inference. Our method favors this development by extending the correlation network by integrating data and knowledge. Especially, two-step extraction of DCGs in Step 1, narrowing down DCGs after filtering DEGs, is a mixture of detecting the significant differences in the gene expression levels and checking the pairwise correlation between gene pairs. This extraction is substitutive to other methods for extracting DCGs, such as WGCNA or gene set net correlation analysis (GSNCA) (Rahmatallah et al., 2014).

### 4.3 Concluding remarks

As a summary of contributions, this study discovered novel *ICAM1*-associated pathways currently absent from the COVID-19 Disease Map. While previous analysis or curation work found the canonical NF-*κ*B pathway (Fujisawa et al., 2021), the non-canonical pathways were not known to be involved in the COVID-19 Disease Map. The discovered pathways suggested the existence of unknown pathways in the map, an upstream non-canonical NF-*κ*B pathway, and a downstream pathway that may lead to MTOC formation subject to observation.

In addition to the scientific findings, our framework, which integrates single-cell omics data analysis and model validation using multiple knowledge bases, is also original and versatile. Especially single-cell omics data analysis in Step 1 and model validation by multiple knowledge bases in Step 3 are realized to construct pathways in different cases (See also Supplementary Material). For these reasons, our work would contribute to a remarkable development in the DD-KB gene network inference methods.

The existence of undirected edges within the final pathways would be a limitation of our framework. These edges without direction arise from correlation networks that find direct and indirect relationships but do not distinguish between causality and correlation (Opgen-Rhein and Strimmer, 2007). Our methodology requires its extension to infer causal directions of the edges.

Consequently, future work will include the following two tasks: one is to infer causal networks based on data and knowledge via Bayesian networks or other observational causal discovery techniques (Pearl, 2000). The other is to analyze the obtained pathways for verifying or modifying them in terms of dynamics. For example, modeling and simulation of differential equations based on state transitions would help us comprehend the dynamics (Odaka and Inoue, 2020). Otherwise, the perturbation experiments can simulate the intervention effects on dynamics by explicitly using direct transcription factor knockout or overexpression (Loucera et al., 2020). Indeed, such a study has significantly improved prediction accuracy for downstream targets (Noh et al., 2018).

Overall, the *ICAM1*-associated pathways constructed from the data and knowledge in this study will expedite the repair and completion of the COVID-19 Disease Map for a deeper understanding of SARS-CoV-2 pathogenesis.

## Data Availability

The original contributions presented in the study are included in the article/Supplementary Material; further inquiries can be directed to the corresponding author.
