# Supplementary material for: Gene network inference from single-cell omics data and domain knowledge for constructing COVID-19-specific ICAM1-associated pathways
Source: Front Genet. 2023 Aug 31;14:1250545. doi: 10.3389/fgene.2023.1250545 (PMC10501835; doi:10.3389/fgene.2023.1250545)
Supplement: Supplementary file 1 [file Image1.PDF]

Supplementary Material

SUMMARY

The purpose of this supplementary material is to provide the detailed results in the main manuscript to help readers better understand the inputs and outputs for each step of the framework. Given this purpose, we display the results in case study 1 only. But any reader who wants to check the figures or datasets in case study 2 can refer to DOI: `10.6084/m9.figshare.23590755`.

Additionally, some readers may find it difficult to read some figures because of the small font size due to the paper size limitation. In that case, they can access the *figshare* link in the caption of each figure to see it in a larger view.

SUPPLEMENTARY FIGURES

The panel labels (a) - (e) in the following supplementary figures stand for infected alveolar type 1 and 2 cells, migratory dendritic cells, tissue-resident alveolar macrophages, monocyte-derived alveolar macrophages, and a summation of all cells.

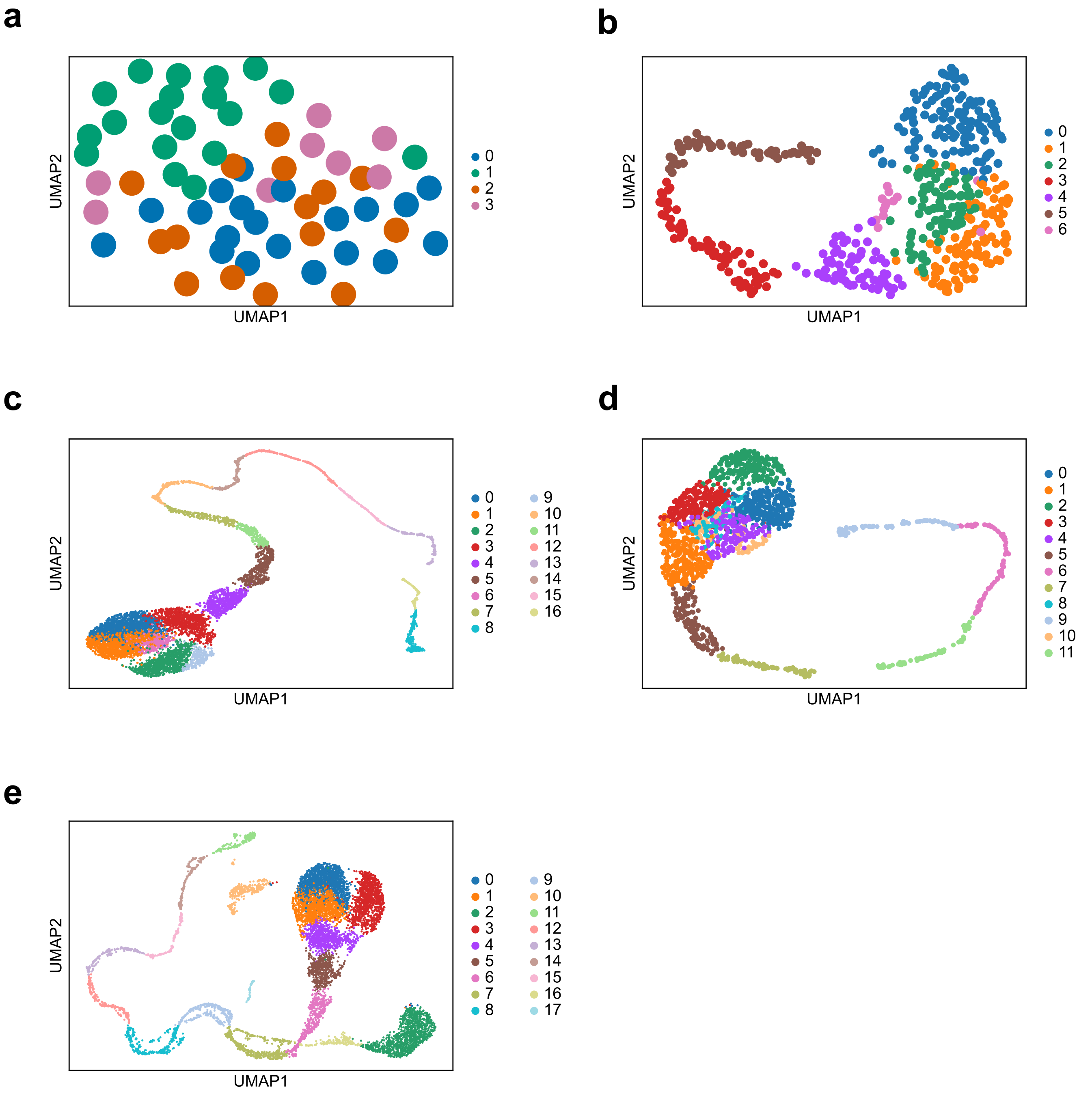

Figure S1. Genewise clustering result. Genewise clustered data points within an embedded latent space. This procedure extracts genes within a specific cluster, including a gene of interest. Euclidean distance measures the distance between clusters. Calculating the log fold change (the magnitude of differential expression) yielded a list of the differential expression genes (DEGs), whose expression levels significantly increased or decreased. Each cluster is assigned a unique cluster number with a different color. See also doi: 10.6084/m9.figshare.17263814.

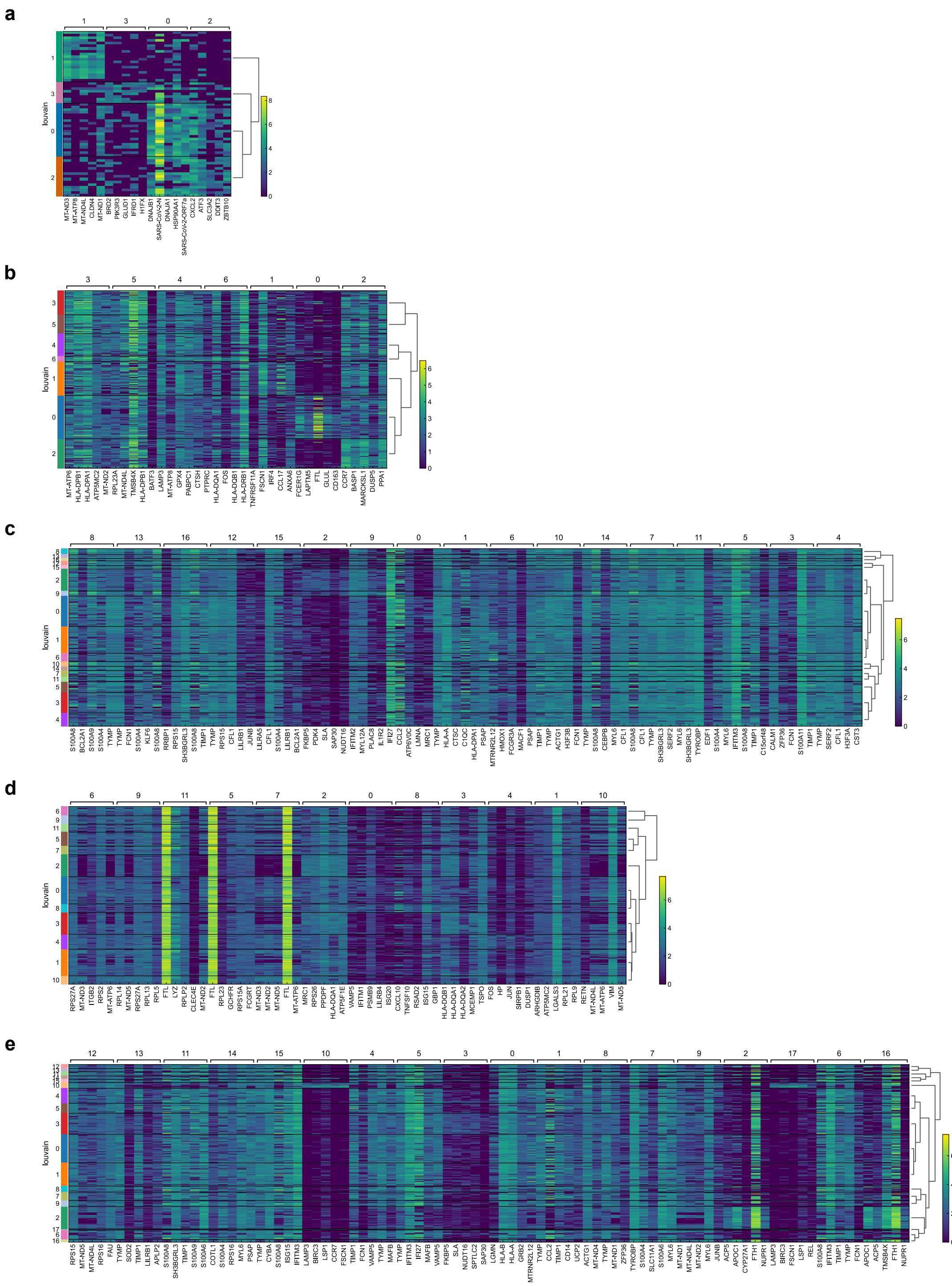

Figure S2. The heatmap of differentially expressed genes. The top five differentially expressed genes (DEGs) for each cluster. Each cluster with a number given by the Louvain algorithm enumerates the corresponding gene names. A brighter color means a higher tendency for differential expression. See also doi: 10.6084/m9.figshare.17263841.

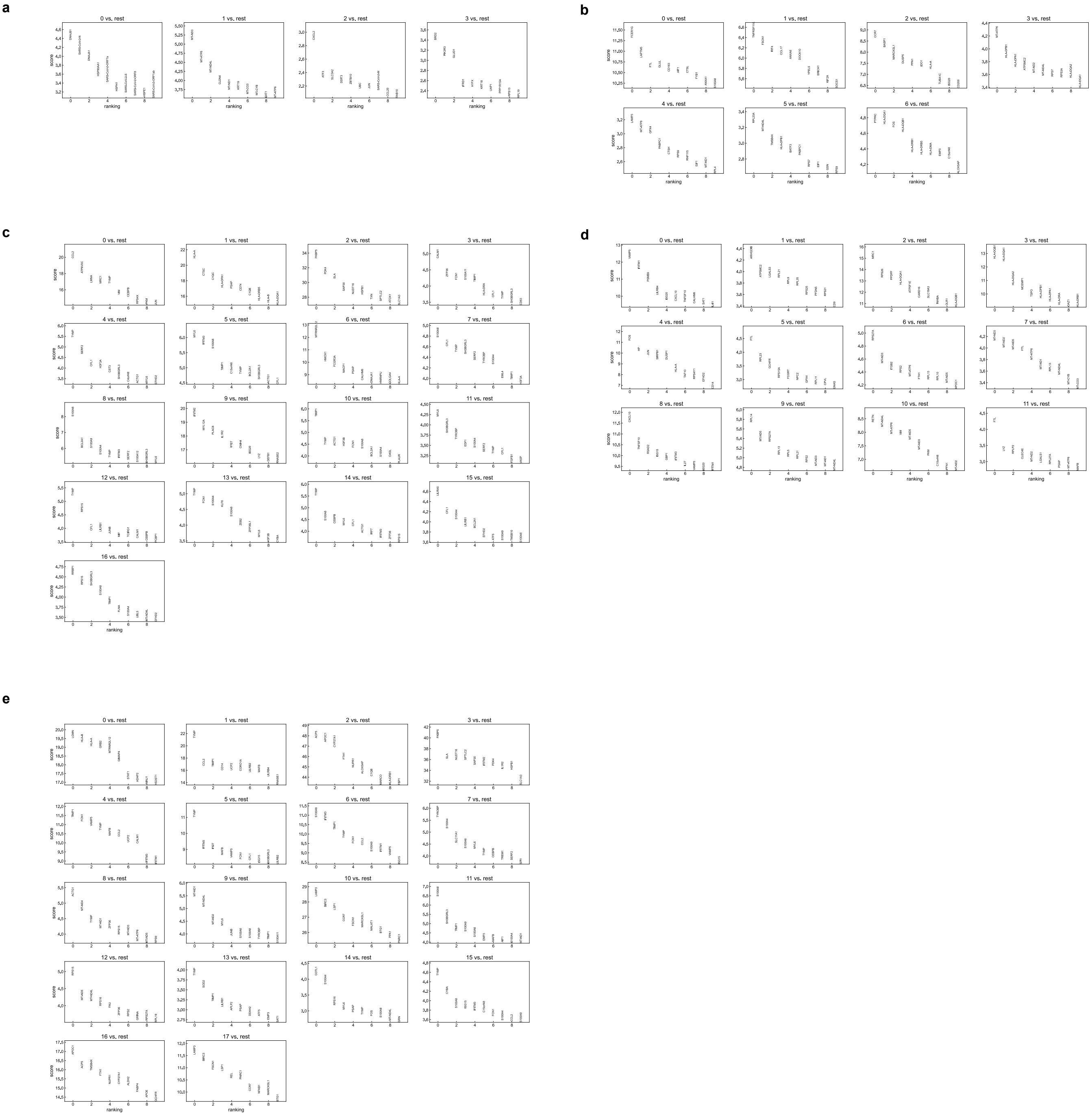

Figure S3. Rank-sum test result for differentially expressed genes. The Wilcoxon rank-sum test determined the clusters' rank-ordered differentially expressed genes (DEGs). Each cluster of each subfigure contains the top 10 genes' names in descending order from left to right, with rank-sum scores assigned to the vertical axes. See also doi: 10.6084/m9.figshare.17263877.

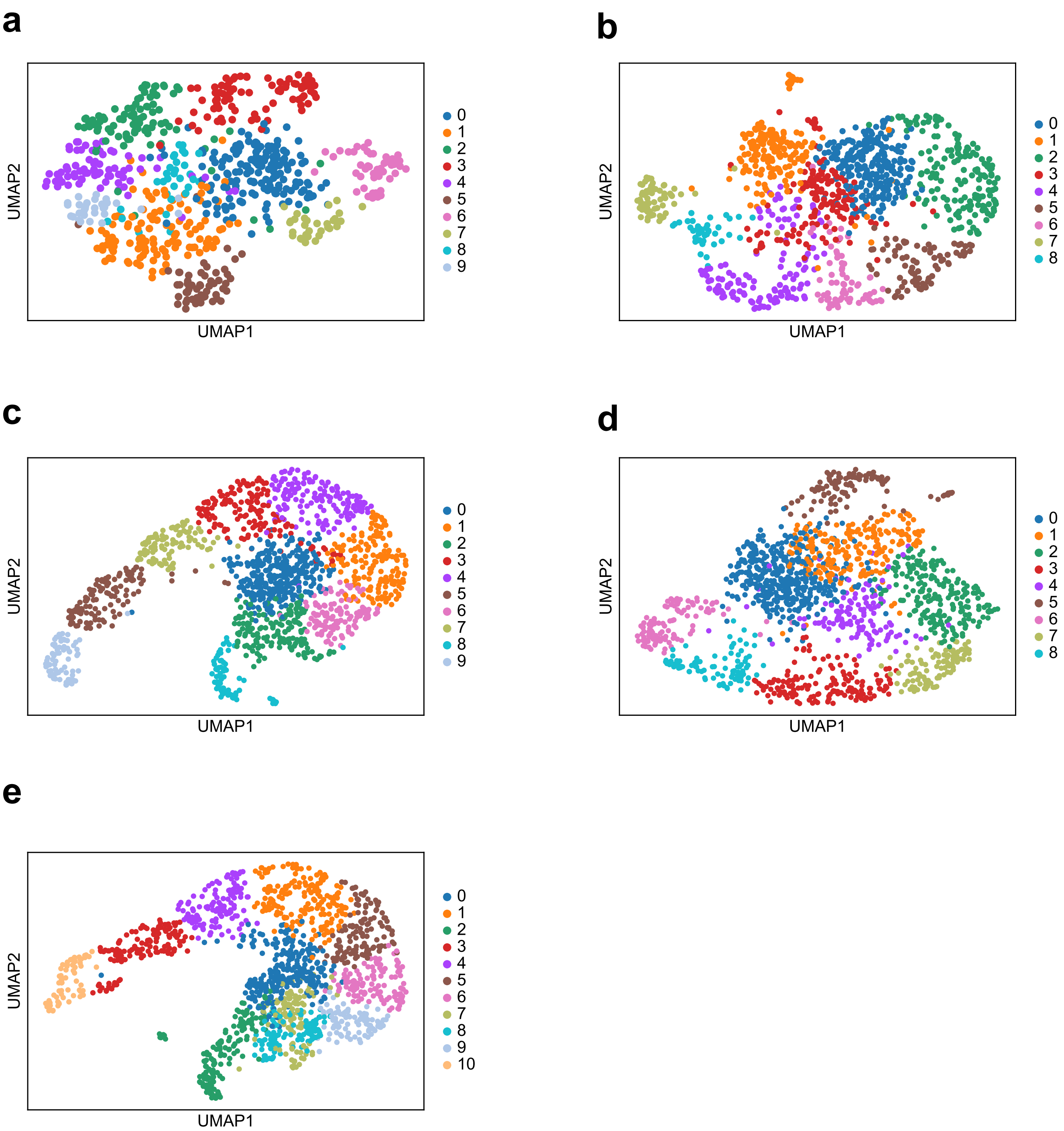

Figure S4. Cellwise clustering result. Cellwise clustered data points within an embedded latent space. Pearson's correlation coefficient measures the distance between clusters. Calculating the log fold change (the magnitude of differential expression) filters a list of the differential coexpression genes (DCGs) from DEGs. Each cluster is assigned a unique cluster number with a different color. See also doi: 10.6084/m9.figshare.17263889.

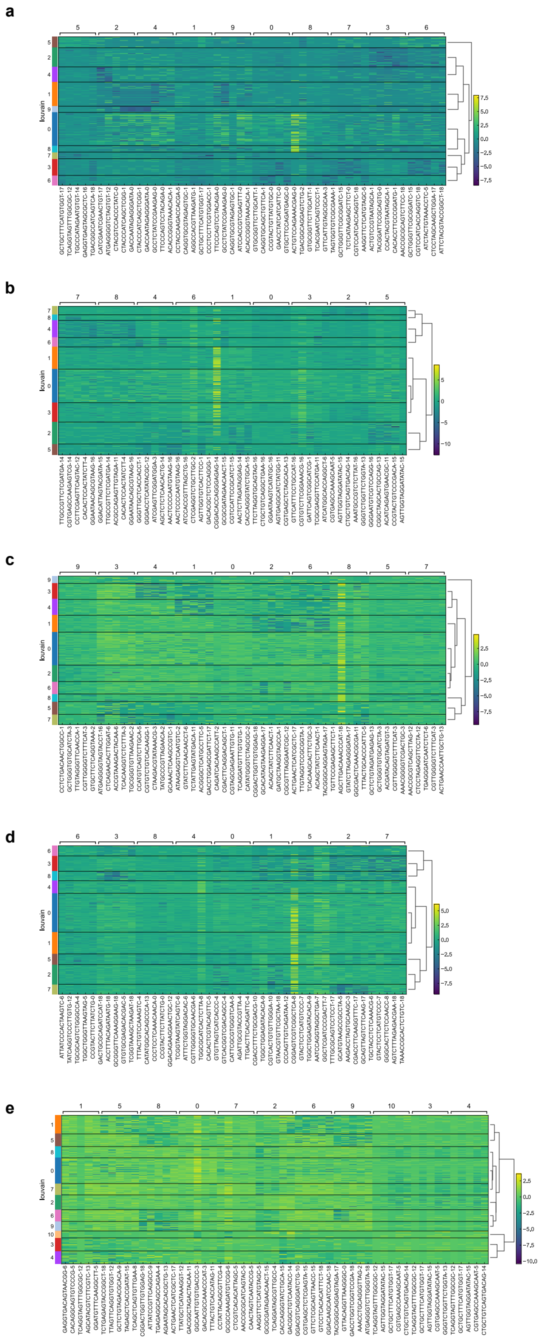

Figure S5. The heatmap of differentially coexpressed genes. Top five cell IDs for each cell cluster with a similar gene coexpression pattern. Each cluster with a number given by the Louvain algorithm enumerates the corresponding cell IDs. A brighter color means a higher tendency for differential coexpression. See also doi: 10.6084/m9.figshare.17263892.

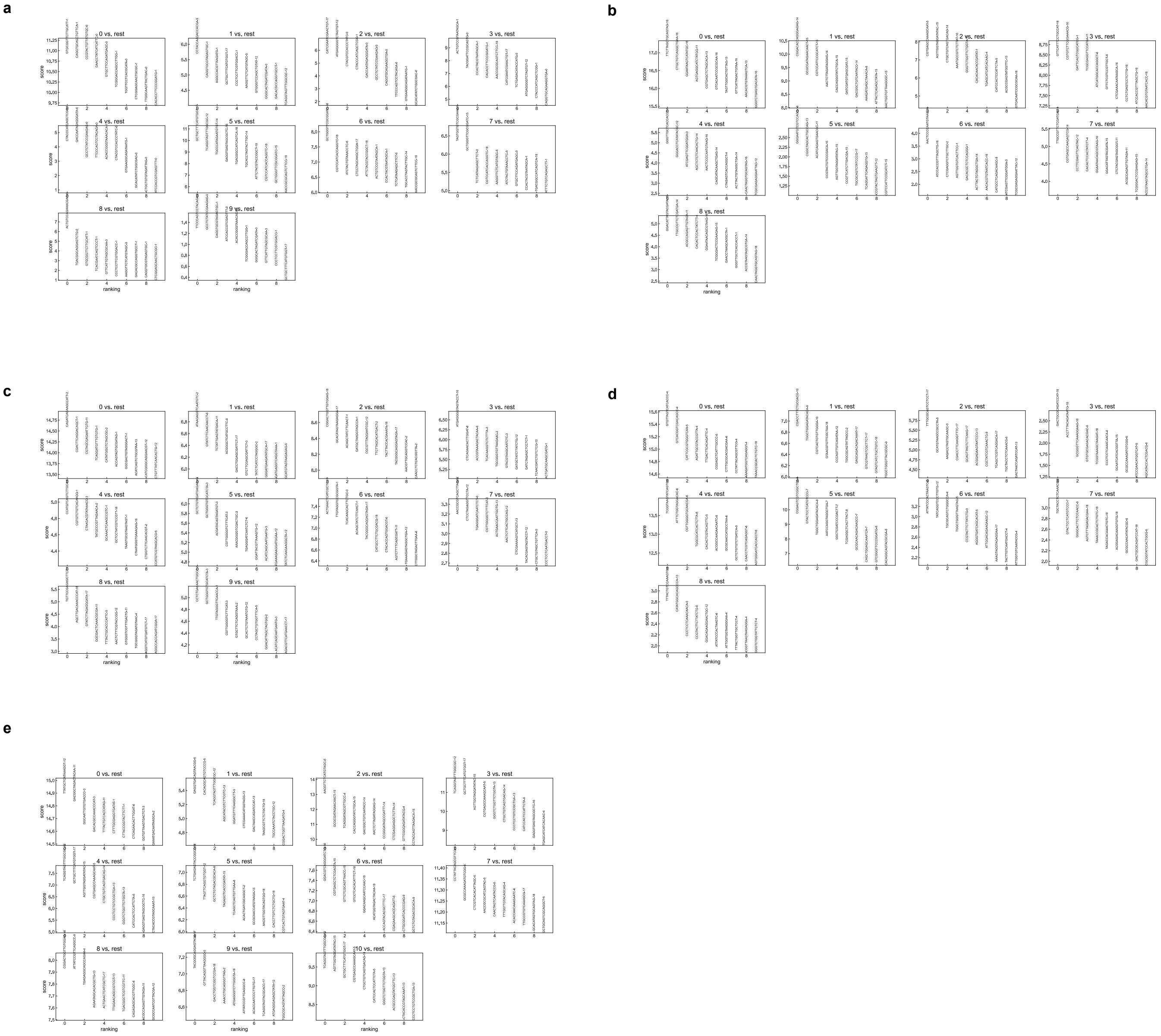

Figure S6. Rank-sum test result for differentially coexpressed genes. Differentially coexpressed genes (DCGs) for each cluster. Each cluster of each subfigure contains the top 10 cells' IDs in descending order from left to right, with rank-sum scores assigned to the vertical axes. See also doi: 10.6084/m9.figshare.17263898.

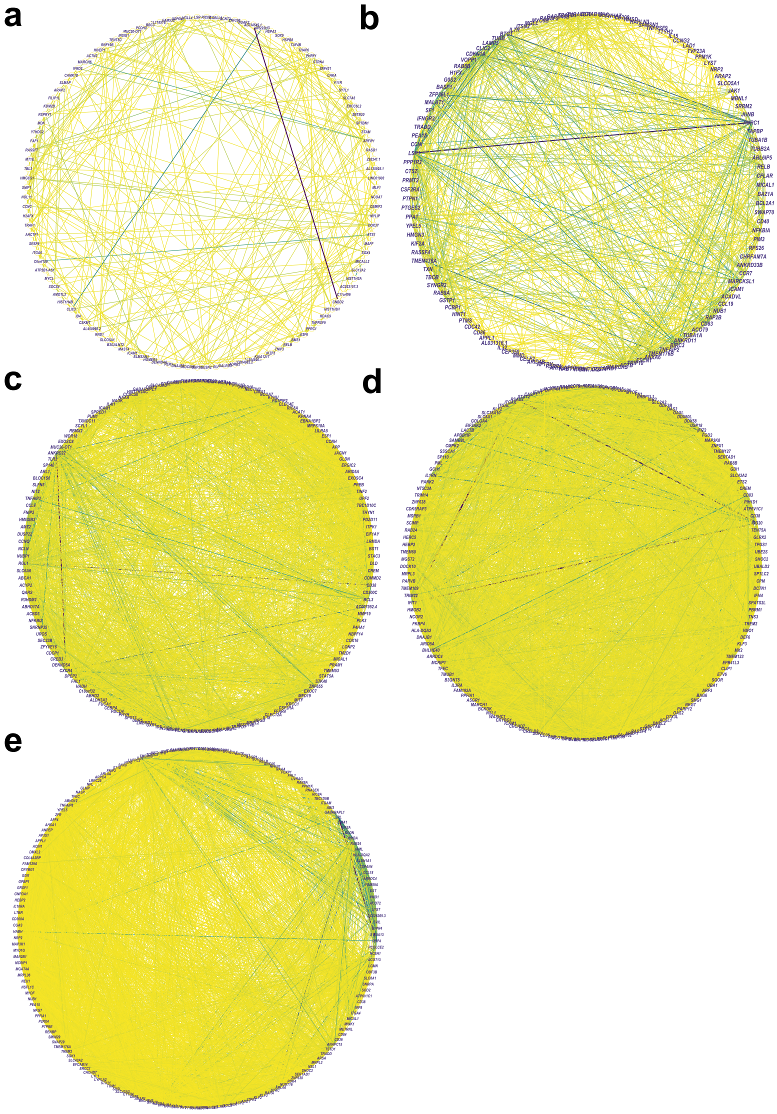

Figure S7. Undirected graphical models. (a) Infected alveolar type 1 and 2 cells; (b) Migratory dendritic cells; (c) Tissue-resident alveolar macrophages; (d) Monocyte-derived alveolar macrophages; (e) All cells. Nodes, edges, and weights are DCGs, relationships between DCGs, and second-order partial correlation coefficients. See also doi: 10.6084/m9.figshare.17261825.

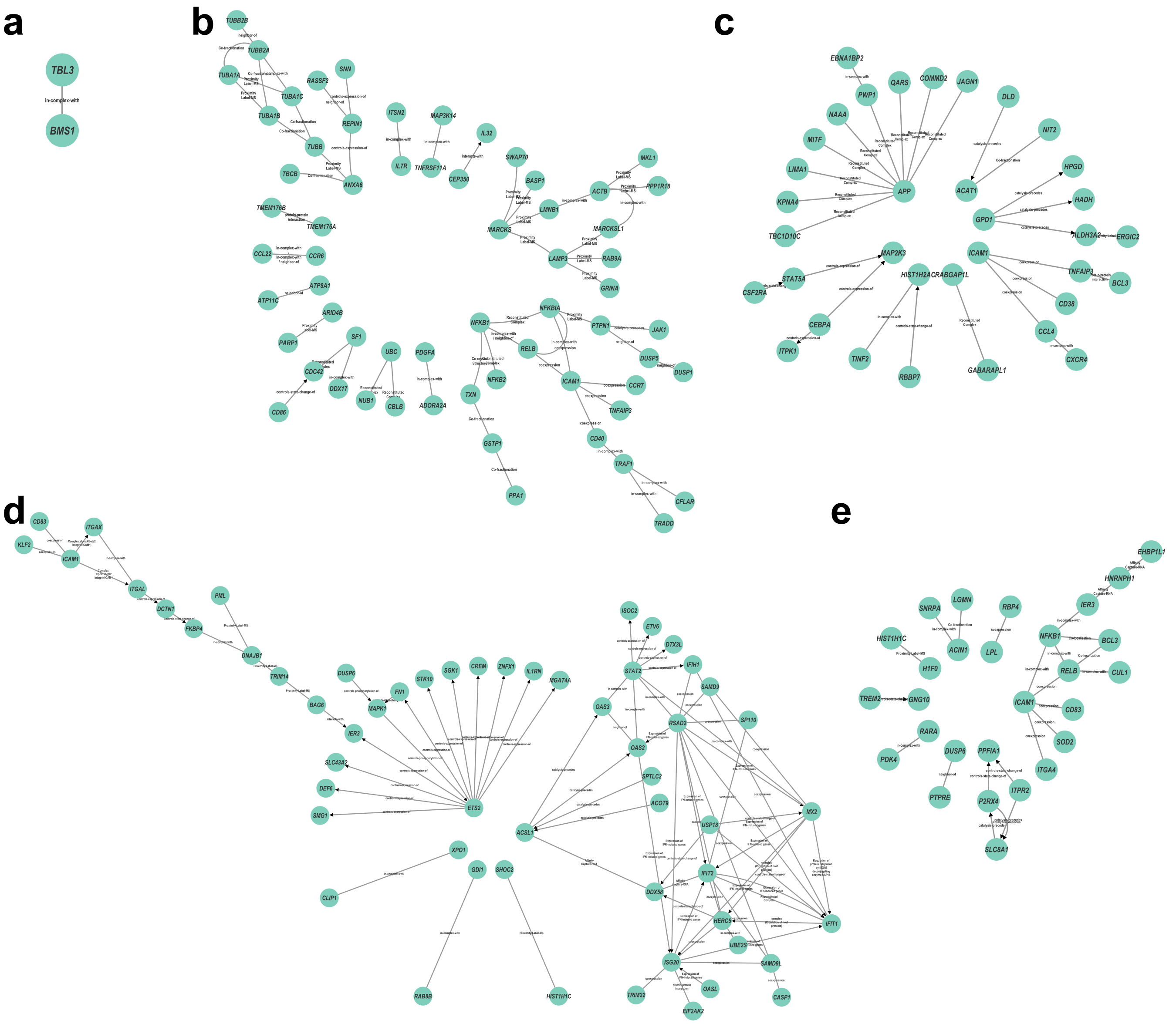

Figure S8. Dependency graphs. (a) Infected alveolar type 1 and 2 cells; (b) Migratory dendritic cells; (c) Tissue-resident alveolar macrophages; (d) Monocyte-derived alveolar macrophages; (e) All cells. Nodes are DCGs, and edges are relationships between DCGs with annotated function names. These function names are validated from knowledge bases. The directed edge is given when the edge is a regulatory relationship, such as activation or inhibition. Undirected edges represent coexpression or other functions without direction. See also doi: 10.6084/m9.figshare.17261780.

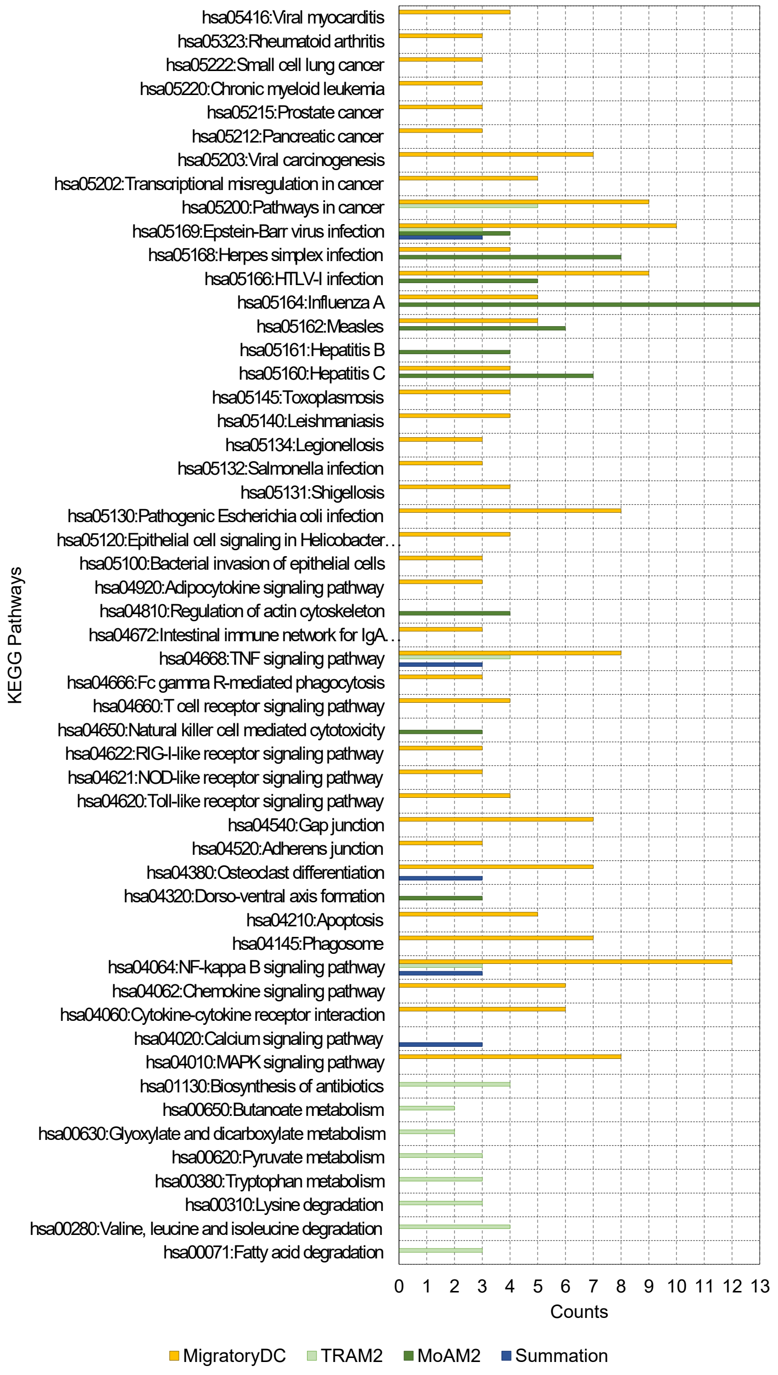

Figure S9. Pathway mapping result. Querying the gene lists of dependency graphs yielded the activated Kyoto Encyclopedia of Genes and Genomes (KEGG) pathways for each cell type. The bars colored yellow, lime green, olive, and navy blue stand for the count of genes that appeared on the KEGG pathways for migratory dendritic cells (DCs), tissue-resident alveolar macrophages (TRAM2), monocyte-derived alveolar macrophages (MoAM2), and all cells. See also doi: 10.6084/m9.figshare.17263907.

SUPPLEMENTARY DATASETS

Differentially expressed genes

The entire list of differentially expressed genes (DEGs). Genewise analysis extracted the gene clusters with differential expression patterns specific to COVID-19. Each cluster includes genes with significant differences in expression from the negative control. Genes within the same cluster share a common differential expression pattern. Signal magnitudes are logarithms of fold changes with cluster identifiers. See also doi: 10.6084/m9.figshare.17273156.

Differentially coexpressed genes

The entire list of differentially coexpressed genes (DCGs). Cellwise analysis filtered the DCGs via three subroutines to classify all the cells into cell clusters based on the correlation coefficients as similarity measurements. Genes within the same cell cluster are more strongly correlated than those in other clusters. Constraining the DCGs with the gene of interest, *ICAM1*, provided a subset of DCGs correlated with *ICAM1*. See also doi: 10.6084/m9.figshare.17273177.

Model validation result

The entire list of relationships of dependency graphs with knowledge bases for model validation. Edge weights include unweighted, weighted unsigned, and weighted signed correlation coefficients. Gray-filled cells in the table are gene pairs that could not be validated based on background knowledge. Therefore, the relations and data sources are missing. The appended tabs marked with (2) contain the tables that exclude unvalidated gene pairs and combine gene pairs with overlapping data sources, i.e., gene pairs contained in two or more different background knowledge. See also doi: 10.6084/m9.figshare.17273120.

APPLYING THE FRAMEWORK TO OTHER GENES OF INTEREST

In case study 2, we applied the framework to not only *ICAM1* but also other genes related to interaction between cells, including *ACTB* and *C15orf48*. Here, *ACTB* encodes β-actin, a non-muscle cytoskeletal filament implicated in cell motility, structure and integrity. *C15orf48* encodes MOCCI (modulator of cytochrome C oxidase during inflammation), which inhibits inflammatory response, as indicated by the downregulation of proinflammatory biomarkers, such as NF-κB, ICAM-1 and VCAM-1 [1]. MOCCI is also likely to cooperate with ICAM-1 and β-actin in COVID-19 for intercellular adhesion.

ACTB-associated pathways

At all three time points, molecules for immune responses such as inflammation or chemotaxis were abundant. For example, Ficolin-1 (*FCN1*) or leukocyte immunoglobulin like receptor (*LILRA5*, *LILRB1*, *LILRB*) were all involved in innate immune responses. As a unique feature of the *ACTB*-associated pathway, the network motif for cell-membrane fusion was conserved. This motif was consisting of the genes encoding major histocompatibility complex (MHC) class II regulating membrane fusion, including *HLA-DPA1*, *HLA-DPB1*, *HLA-DRA*, *HLA-DRB1*, *HLA-DRB5*, and *HLA-DQB1*. Relevant to MHC class II, HLA class II histocompatibility antigen γ chain (*CD74*) involved in the formation of MHC class II peptide complexes for CD4+ T cell responses or Cathepsin S (*CTSS*) related to antigen presentation with MHC class II were present. Moreover, Vimentin (*VIM*) for cytoskeleton formation or actin-binding protein allograft inflammatory factor 1 (*AIF1*) were identified.

C15orf48-associated pathways

Throughout the three-time points, molecules for immune response or macrophage polarization were apparent. There were also similar features with the *ICAM1*-associated pathways. For instance, both *ICAM1* and *C15orf48* pathways contained C-X-C Motif Chemokine Ligand (*CXCL*), interleukin 1 β (*IL1B*), transmembrane protein 176A/B (*TMEM176A*, *TMEM176B*), TNF-α-induced protein 6 (*TNFAIP6*), interleukin 1 receptor antagonist (*IL1RN*).

[1] Lee, C.Q.E., Kerouanton, B., Chothani, S. et al. Coding and non-coding roles of MOCCI (C15ORF48) coordinate to regulate host inflammation and immunity. *Nat. Commun.* 12, 2130 (2021). doi:10.1038/s41467-021-22397-5

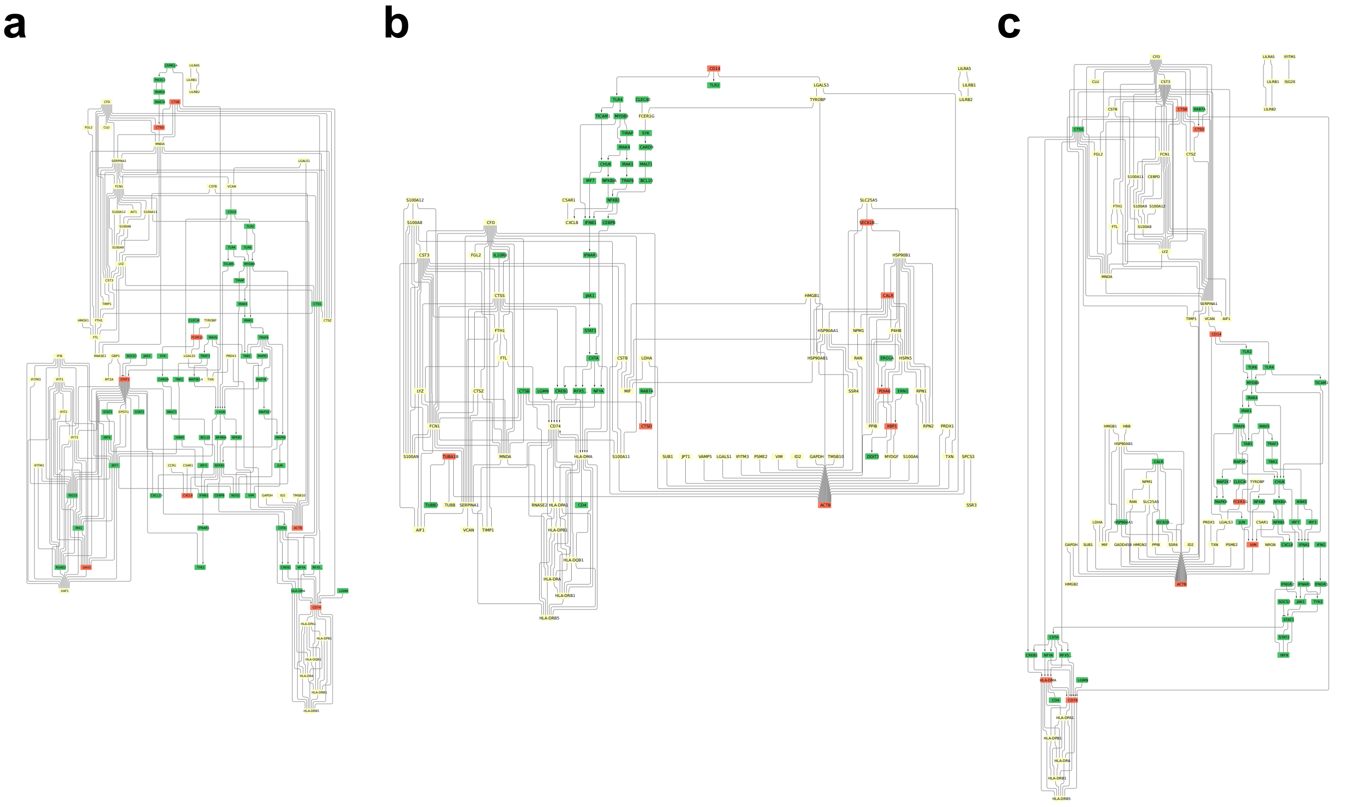

Figure S10. *ACTB*-associated pathways at different time points **(a)** Day1, **(b)** Day 5, and **(c)** Day 10. The figures and datasets regarding *ACTB*-associated pathways can be referred in *figshare*: 10.6084/m9.figshare.23591490.

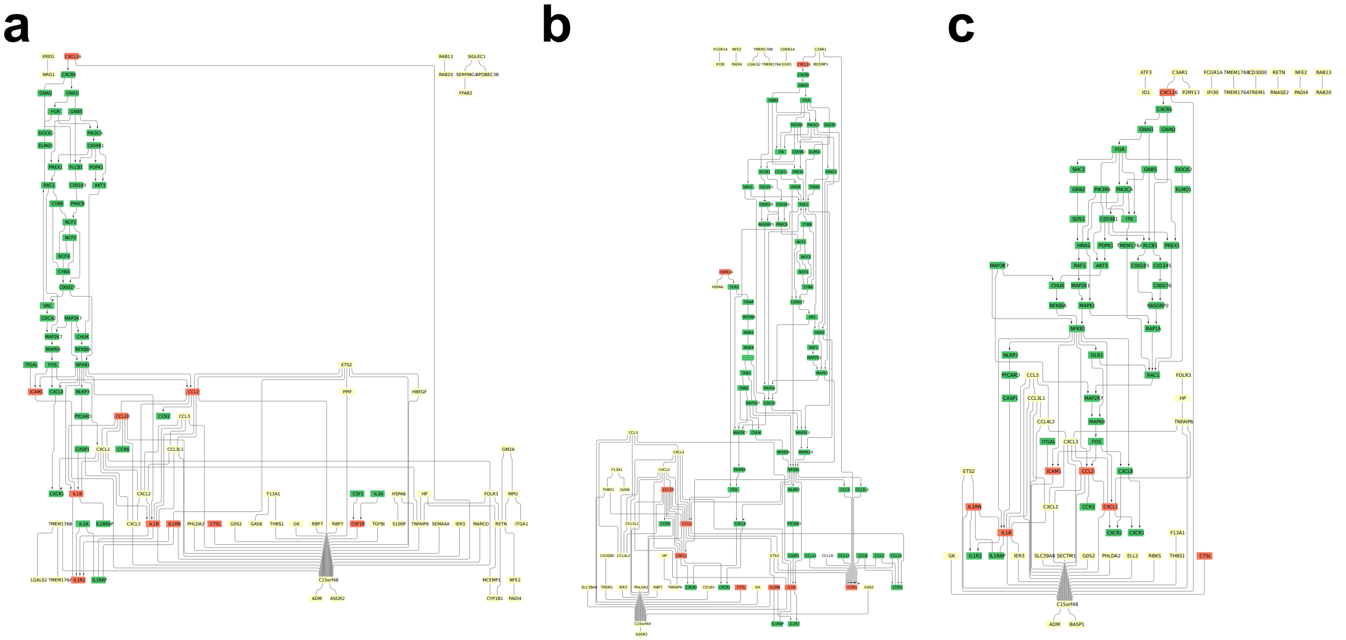

Figure S11. *C15orf48*-associated pathways at different time points **(a)** Day1, **(b)** Day 5, and **(c)** Day 10. The figures and datasets regarding *C15orf48*-associated pathways can be referred in *figshare*: 10.6084/m9.figshare.23591505.

TIME-DEPENDENT CHANGE OF THE THREE PATHWAYS

To grasp the time-dependent change of activated pathways, we drew the parallel coordinates for each matched KEGG and Reactome pathway, ranking in descending order of the gene counts in the pathways. Here, the Reactome pathway is an alternative pathway database that includes information more about pathological names than about specific disease names that the KEGG pathway tends to label.

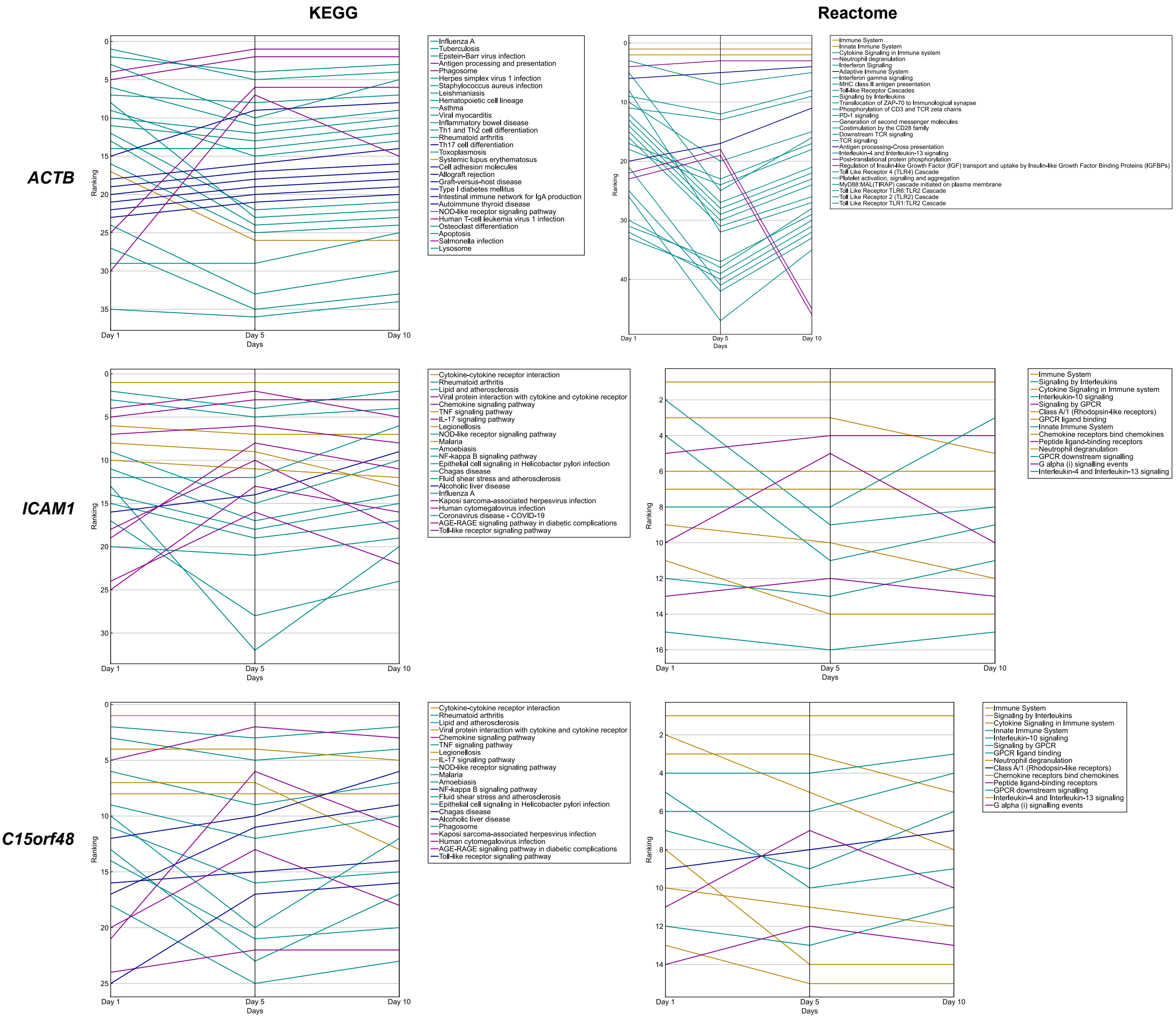

Figure S12. Parallel coordinate ranking plots of the matched KEGG and Reactome pathways. See this figure for a larger view in *figshare*: 10.6084/m9.figshare.23576238.

In the figure, the different line colors indicate the variation of temporal pattern over the three-time points. Purple, green, yellow, and dark blue colored lines reflect an increase followed by a decrease in the number of hit genes, a decrease followed by an increase, a monotonic decrease, and a monotonic increase.

Comparison between the three ranking plots tells us that the inferred pathways are plausible in terms of the COVID-19 typical clinical time courses such as innate immunity or neutrophil degranulation. In

addition to that, both in *ICAM1*-associated pathways and *C15orf48*-associated pathways, signaling by G-Protein Coupled Receptor (GPCR) and Class A/1 (Rhodopsin-like receptors) are found. Considering that GPCR largely includes the Rhodopsin-like family proteins and they regulates microtubule stabilization[2], we cannot deny that microtubule organizing center (MTOC) formation and viral cell-to-cell transmission could be observed in COVID-19 as well. Verifying the MTOC formation or cell-to-cell transmission in COVID-19 would require further *in vitro* infection experiments with microscopy.

[2] Palazzo A.F., Joseph H.L., Chen Y.J., et al. Cdc42, dynein, and dynactin regulate MTOC reorientation independent of Rho-regulated microtubule stabilization. *Curr. Biol.* 2001;11(19):1536-1541. doi:10.1016/s0960-9822(01)00475-4
